# Factors and at-risk group associated with hypertension self-management patterns among people with physical disabilities: a latent class analysis

**DOI:** 10.1186/s12889-022-13482-5

**Published:** 2022-05-25

**Authors:** Hye Jin Nam, Ju Young Yoon

**Affiliations:** 1 College of Nursing, Seoul National University, Seoul, 03080 South Korea; 2 Research Institute of Nursing Science, Seoul National University, Seoul, 03080 South Korea; 3 Center for Human-Caring Nurse Leaders for the Future by Brain Korea 21 (BK 21) Four Project, College of Nursing, Seoul National University, Seoul, 03080 South Korea

**Keywords:** Latent class analysis, Physically disabled, Hypertension, Self-management

## Abstract

**Background:**

People with disabilities are vulnerable to chronic diseases such as hypertension. In South Korea, over half of the population living with a physical disability suffer from hypertension. Understanding the typology of hypertension self-management patterns will assist with behavioural interventions for people with physical disabilities. Thus, this study aims to identify the typology of hypertension self-management behavioural patterns, the factors associated with the latent classes, and to recognise potential at-risk populations by comparing potential health outcomes among hypertensive adults with physical disabilities.

**Methods:**

Data of 1551 participants were extracted from the 2017 National Survey of Disabled Persons. Latent classes were analysed using five indicators of self-management: smoking, alcohol consumption, physical activity, diet, and weight control. Determinants of self-management patterns, such as general characteristics, health-related factors, and social relationships, were identified using multinomial logistic regression. Further, health measures, such as health profile, psychological health, and patient experience, were compared.

**Results:**

The following three latent classes were identified: “high self-management” group (40.8%), “harmful habitual behaviour” group (20.6%), and “inactive behaviour” group (38.6%). Compared with the high self-management group, the predictors of belonging to the harmful habitual behaviour group were being male, young, and single. Being female, employed, severely disabled, dependent, and unsatisfied with friendships were predictors of the inactive behaviour group. Those in the inactive behaviour group had a poor health-related quality of life, poor subjective health, depression, and unmet medical needs.

**Conclusions:**

This study provides evidence that there are mutually exclusive subgroups of patients with hypertension regarding self-management patterns, identifies an array of predictive factors in each latent class membership, and distinguishes a high-risk group by comparing the health measures among patients with hypertension with physical disabilities. Analysing subgroups may assist in identifying and meeting the diverse needs of self-management support in hypertensive patients with physical disabilities.

## Introduction

Hypertension is a major risk factor for cardiovascular disease and premature death. The World Health Organization estimates that hypertension affects more than one billion individuals worldwide and accounts for 13.5% of total deaths per year [1]. Serious concerns about hypertension in people with disabilities have been regularly reported in national studies. In South Korea, 56.4% of the population living with a physical disability was affected by hypertension in 2017, which was twice as high as the population without a disability [2]. People with disabilities have been shown to are at 2.3-fold high risk of developing hypertension and five times higher mortality rates due to hypertension than the general population [3, 4]. Statistical reports have consistently identified people with disabilities as experiencing poorer health outcomes, resulting in a higher mortality rate than their peers in the general population. Unfortunately, to date, most studies have focused mainly on hypertension in the general population, whereas the subpopulation that experiences disabilities has largely been neglected.

Engagement in self-management behaviour is indispensable for the management of hypertension. The 2018 Korean Society of Hypertension Guidelines provide self-management recommendations essential for the control of high blood pressure including abstaining from smoking, limiting alcohol consumption, eating a diet rich in fruits and vegetables, taking part in regular physical activity, and maintaining a healthy weight [5]. Adherence to such self-management behaviours significantly reduces blood pressure, resulting in more cost-effective use of healthcare resources [6]. The main causes of mortality in hypertensive patients are linked to unhealthy behaviours such as physical inactivity, tobacco smoking, poor diet, obesity, and excessive alcohol consumption [7, 8]. Overwhelming substantive evidence supports the significant role of self-management behaviour on blood pressure [9].

Despite the clear benefits of self-management behaviours, many hypertensive patients do not comply with the suggested recommendations, thereby failing to regulate their blood pressure. Self-management may be even more challenging for those with physical disabilities due to their complex needs, such as physical restrictions and co-morbid health conditions, thus highlighting the need for extra support and appropriate accommodations [10]. Providing safe, appropriate, and accessible options for people with disabilities requires support across many sectors to help them engage more easily in essential healthy behaviours [11]. However, there is a lack of scientific evidence that supports addressing priority health needs and the identification of key determinants of health behaviours in people with physical disabilities.

A diverse array of characteristics and physical needs has been demonstrated among people with physical disabilities [12]. Thus, it is critical to take special consideration of the unique needs of individuals with physical disabilities in relation to self-management behaviours. Exploration of person-centred typologies could contribute to understanding the heterogeneity of self-management behaviours. Thus far, however, it remains unclear how the different aspects of hypertension management regimes appear in behavioural patterns among people with physical disabilities. To date, the majority of studies have focused only on hypertension management behaviour as a single concept, and the level of practice was evaluated by a total score of unidimensional measure [7]. This traditional variable-level method has limitations as it has led to reduced appreciation of the diversity of patient challenges and specific needs, which has then fostered misleading and overgeneralised conclusions with study findings representing the overall sample [13]. This methodological limitation of traditional variable-centred methods may be avoided by utilising a latent class analysis (LCA) approach. LCA is a person-centred method, suitable for discrete and dichotomous variables; it also enables the identification of distinct configurations of heterogeneity within a given population sample [13]. Hence, LCA has been described as a more logical and informative approach for exploring health behaviours [7, 13].

Therefore, this study aimed to identify the typology of hypertension self-management behaviours to improve understanding of self-management patterns using LCA on the basis of the clustering of five hypertensive management recommendations: abstaining from smoking, limiting alcohol consumption, adequate physical activity, balanced diet, and weight control. We also aimed to explore the factors associated with these behaviours and how these classes differ with respect to various health measures to identify potential unobserved, at-risk groups among hypertensive adults with physical disabilities living in South Korea.

## Methods

### Study population

This cross-sectional study analysed data from the 2017 National Survey of Disabled Persons, a nationally representative survey of community-dwelling people with disabilities in South Korea [2]. This survey is conducted by the Ministry of Health and Welfare and the Korea Institute for Health and Social Affairs every 3 years, in order to provide nationally representative data and descriptive statistics of people with disabilities. It utilises a two-stage, stratified random cluster sampling method drawn from the 2015 Population and Housing Census of Korea. The inclusion criteria for this study were those patients who have a physical disability and were diagnosed with hypertension. Physical disability refers to physical limitations that result from a permanent functional disorder in a physical body part such as muscle, nervous system, or bone structure, whether the patients acquired the disability either congenitally or postnatally [14]. Consequently, 1551 eligible participants were extracted from the dataset of 6549 patient samples.

### Ethical considerations

This study was a secondary analysis of cross-sectional survey data. The 2017 National Survey of Disabled Persons has stringent protocols that ensure confidentiality and participants’ autonomy. Written informed consent was obtained from all subjects or their legal guardians. To ensure compliance with de-identified data handling procedures, our study was approved by the Institutional Review Board of Seoul National University (IRB No. E2011/002–013).

### Measurements

#### Latent class indicators

Five hypertension self-management indicators were selected: cigarette smoking, alcohol consumption, physical activity, diet, and weight control. Binary indicators, “yes” versus “no,” for each self-management behaviour were created based on existing recommendations of 2018 Korean Society of Hypertension Guidelines [5] as described below.

##### Cigarette smoking

The 2018 Hypertension Guidelines emphasise that smoking cessation is strongly recommended as smoking is a major risk factor for hypertension [5]. The answers to a single question asking “Do you smoke?” were recoded as a binary variable. Thus, the responses “never smoked” and “former smokers” were classified as “yes,” and “daily smoker” and “occasional smoker” were recoded as “no” in regards to whether or not the participant adhered to the 2018 Hypertension Guidelines.

##### Alcohol consumption

Excessive alcohol intake can elevate blood pressure and resistance to antihypertensive drugs. Thus, it is recommended that hypertensive patients drink less than two glasses of alcohol per day [5]. The participants who did not drink alcohol within the last year and those who reported “1–2 glasses” were recoded as “yes” and those who drank “3–4 glasses” or more were classified as “no” in regards to whether or not the participant adhered to the 2018 Hypertension Guidelines.

##### Physical activity

Physical activity can contribute to the control of high blood pressure and deter complications. The 2018 Hypertension Guidelines recommend regular physical activity for > 30 min a day at least three times per week for hypertension management [5]. Those who reported that they engaged in physical activity for 30 or more minutes at a time, more than three times a week were classified as “yes”; others were classified as “no.”

##### Diet

Eating a low-fat diet rich in whole grains, vegetables, and fruits and contains adequate nutrients can help control high blood pressure [5]. Participants were asked, “During the last week, have you consumed a nutritionally-balanced diet?” Those who answered “yes” to the question were considered as adherent to dietary recommendations.

##### Weight control

Body weight control is suggested to avoid obesity, which is a risk factor for uncontrolled high blood pressure. It is recommended that Body Mass Index (BMI) be maintained at 25 kg/m [5]. BMI values were calculated by dividing the participant’s weight in kilograms by the square of their height in metres. This variable was categorised as “yes” for a BMI of ≤25 kg/m^2^, as adhering to the 2018 Hypertension Guidelines and “no” if ≥25 kg/m^2^.

#### General characteristics

General characteristics of the participants included demographics, health-related variables, and social relationships. Demographic characteristics such as age, gender, education level, marital status, employment status, and monthly household income as well as health-related variables such as disability location and severity, ability to perform activities of daily living (ADL) and/or instrumental ADL (IADL), and the number of comorbidities were recorded. Household income was calculated based on equivalised income (i.e., total household income divided by the square root of the number of household members) and classified as higher or lower based on the median income. Disability severity was assessed by asking about the degree of disability as registered in the Disability Registration System; missing data (*n* = 23) was discovered because some participants had not been officially registered. The responses regarding ADL and IADL were categorised into “dependent” if the participant required assistance with one or more activities. Quality of social relationships was assessed using satisfaction with relationships with friends and family.

#### Health measures

Health profiles were measured using the Health-Related Quality of Life (HRQOL) scale and participants’ subjective experience of their health. The EuroQOL-5 Dimension (EQ-5D) was used to assess the HRQOL. Value sets developed by Lee et al. [15] were applied to the data to convert the states derived from the EQ-5D into an index score. Subjective health was assessed using a single question “How do you feel about your health in general?” Psychological health was measured using suicidal ideation, suicide attempts, and depression. Suicidal ideation and suicide attempts were assessed using questions “Have you ever felt suicidal within the last year?” and “Have you ever attempted suicide within the last year?”, respectively. Depression was assessed by asking “Have you ever felt sad or desperate enough such that it interfered with your daily life for more than two weeks in a row within the last year?” and requesting a dichotomous response. Patient experiences with the healthcare system were evaluated using satisfaction with healthcare services and unmet medical needs. Patient satisfaction consisted of a single item that questioned the degree of satisfaction with the healthcare services that the participants used recently. Unmet medical needs were assessed by asking whether the participants experienced being unable to use the hospital or healthcare services when they needed to during the last year.

### Statistical analysis

Data analysis was conducted using IBM SPSS Statistic 21.0 (IBM Corp., Armonk, NY, USA) and Mplus 8.0 (Muthén and Muthén, Los Angeles, CA, USA). LCA was conducted using five indicators: cigarette smoking, alcohol consumption, physical activity, balanced diet, and weight control; all variables were considered binary categories. The analytical strategy in LCA involves identifying the statistically optimal number of classes that explain hypertension self-management behavioural patterns across the five indicators. The optimal number of classes was determined by progressively increasing the number of classes and comparing the model selection statistics of each subsequent model [16]. Missing data were handled using full information maximum likelihood.

Model comparisons were performed using the measures of model-fit indices, including Akaike’s Information Criteria (AIC), Bayesian Information Criterion (BIC), the adjusted BIC (saBIC), entropy, and the Lo-–Mendell–Rubin Likelihood Ratio test (LMR). Smaller values of AIC, BIC, and saBIC suggest a better, more parsimonious model and inform the decision on the best model to be retained [7]. A significant *p*-value on the LMR (*p* < .05) indicates a model with good fit [16]. Entropy calculations close to 1.0 indicate a better classification [16]. In addition to the model fit, we reviewed the substantive meaning of latent class models.

Descriptive statistics, such as frequencies, percentages, means, and standard deviations, were used to describe the general characteristics of the total sample. Once the appropriate number of latent classes was determined, the characteristics of each group and health measures were compared using a chi-squared test and analysis of variance with Duncan’s test for significance. The demographics and health-related variables were compared among emergent latent classes using multinomial latent class logistic regression. Missing values were estimated using multiple imputations.

## Results

### Latent class model of hypertension self-management

Of the 6549 people with disabilities who participated in the 2017 National Survey of Disabled Persons, 1551 people were included in the study. To determine the latent classes for hypertension self-management behaviour, two-, three-, four-, and five-latent class models were tested as shown in Table 1. Although a two-latent class model exhibited the lowest BIC value, a three-latent class model was selected based on the values of AIC, saBIC, entropy, and the *p*-value of the LMR as the overall values indicated that it was the parsimonious, best-fitting model. The entropy of the three-latent class model was 0.74, which exceeded the criteria for a good class separation (> 0.60) [16].

**Table 1:** Comparison of latent class analysis models with different latent classes based on model selection statistics

Category	Class 2	Class 3	Class 4	Class 5
AIC	8873.32	8859.62	8854.69	8859.10
BIC	8932.13	8950.51	8977.66	9015.05
saBIC	8897.19	8896.50	8904.59	8922.92
Entropy	0.58	0.74	0.48	0.83
LMR	176.26*	25.13*	16.55	7.48

*p < .05

*AIC* Akaike’s information criterion, *BIC* Bayesian Information Criterion, *saBIC* the adjusted BIC, *LMR* Lo–Mendell–Rubin likelihood ratio test

**Fig. 1:**
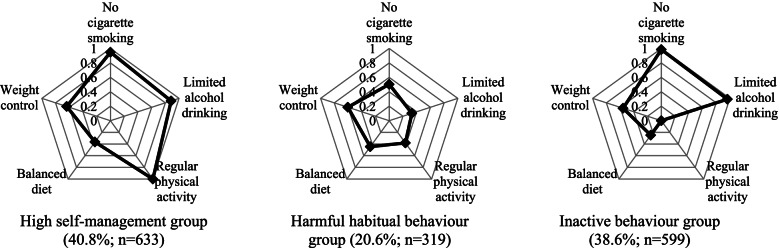
Probabilities of five hypertensive self-management indicators estimated in three latent classes

The latent class membership and response probabilities for the five self-care behaviours are detailed by each of the latent classes in Table 2. Figure 1 also illustrates the magnitude of the discrepancy between the three latent classes by visualising the response probabilities for each indicator. The latent classes 1, 2, and 3 were labelled as the “high self-management” group, the “harmful habitual behaviour” group, and the “inactive behaviour” group, respectively, based on the probabilities of hypertension self-management behaviours from each latent class.

**Table 2:** Estimated classes and response probabilities from a three class model of hypertension self-management behaviour in patients with physical disabilities

Variables	Category	Total	High self-management group	Harmful habitual behaviour group	Inactive behaviour group
Probability of latent class membership		100% (*N* = 1551)	40.8% (class 1; *n* = 633)	20.6% (class 2; *n* = 319)	38.6% (class 3; *n* = 599)
No cigarette smoking	Yes	0.852	0.949	0.504	0.985
	No	0.148	0.051	0.496	0.015
Limited alcohol consumption	Yes	0.778	0.885	0.331	0.964
	No	0.222	0.115	0.669	0.036
Physical activity	Yes	0.467	1.000	0.381	0.000
	No	0.533	0.000	0.619	1.000
Balanced diet	Yes	0.340	0.365	0.446	0.248
	No	0.660	0.635	0.554	0.752
Weight control^a^	Yes	0.562	0.638	0.603	0.557
	No	0.375	0.362	0.397	0.443

^a^Missing values included using full information maximum likelihood

The high self-management group (Class 1; 40.8%; n = 633) consisted of hypertensive adults with physical disabilities who had relatively high probabilities of all five self-management indicators (non-smoking: 94.9%; limited alcohol consumption: 88.5%; balanced diet: 36.5%; and weight control: 63.8%). Noticeably, all of the participants in this group were adherent to regular physical activity (100.0%).

The harmful habitual behaviour group (Class 2; 20.6%; *n* = 319) exhibited the lowest probabilities of being a non-smoker (50.4%) and of limiting alcohol consumption (33.1%). They had moderate probabilities of practicing regular physical activity (38.1%), having a balanced diet (44.6%), and weight within the normal range (60.3%).

The inactive behaviour group (Class 3; 38.6%; *n* = 599) had the highest probabilities of non-smoking status (98.5%) and limited alcohol drinking (96.4%). Conversely, they had the lowest probabilities of having a balanced diet (2.4%) and proper weight control (55.7%), and no one from this group engaged in regular physical activity (0.0%).

### Participants’ characteristics

Table 3 displays the general characteristics of all participants, as well as the comparisons of the characteristics between the three latent classes. Results showed that gender (*p* < .001); age (*p* < .001); education (*p* < .001); marital status (*p* = .002); employment status (*p* < .001); monthly household income (*p* < .001); area of physical disability such as upper limbs (*p* < .001), lower limbs (*p* = .001), or spine (*p* = .001); disability severity (*p* < .001); ADL (*p* < .001) and/or IADL (*p* < .001) dependent status; the number of comorbidities (*p* < .001); and satisfaction with friendships (*p* < .001) were significantly different among the three classes.

**Table 3:** Characteristics of total participants and comparisons between the latent classes in hypertensive patients with physical disabilities

Variables	Category	Total (*N* = 1551)		High self-management group (class 1; *n* = 633)		Harmful habitual behaviour group (class 2; *n* = 319)		Inactive behaviour group (class 3; *n* = 599)		*×*^2^ or F^a^	*p*	Post hoc^b^
		n (%) or M (SD)										
Gender	Male	722	(46.6)	270	(42.7)	286	(89.7)	166	(27.7)	327.51	<.001	
	Female	829	(53.4)	363	(57.3)	33	(10.3)	433	(72.3)			
Age (years)		69.88	(10.28)	71.17	(8.77)	61.95	(10.80)	72.75	(9.32)	146.35	<.001	2 < 1 < 3
Education	No education	301	(19.4)	119	(18.8)	20	(6.2)	162	(27.0)	163.81	<.001	
	Elementary school	609	(39.3)	267	(42.2)	83	(26.0)	259	(43.2)			
	Middle school	260	(16.8)	107	(16.9)	64	(20.1)	89	(14.9)			
	High school	283	(18.2)	105	(16.6)	108	(33.9)	70	(11.7)			
	≥ University	98	(6.3)	35	(5.5)	44	(13.8)	19	(3.2)			
Current marital status	Married	889	(57.3)	370	(58.5)	205	(64.3)	314	(52.4)	12.45	.002	
	Single	662	(42.7)	263	(41.5)	114	(35.7)	285	(47.6)			
Employment status	Employed	559	(36.0)	187	(29.5)	185	(58.0)	187	(31.2)	84.33	<.001	
	Unemployed	992	(64.0)	446	(70.5)	134	(42.0)	412	(68.8)			
Monthly household income^c^	Low	777	(50.1)	335	(52.9)	103	(32.3)	339	(56.6)	52.60	<.001	
	High	774	(49.9)	298	(47.1)	216	(67.7)	260	(43.4)			
Disabled body part	Upper limbs	315	(20.3)	118	(18.6)	102	(32.0)	95	(15.9)	35.24	<.001	
	No	1236	(79.7)	515	(81.4)	217	(68.0)	504	(84.1)			
	Lower limbs	818	(52.7)	309	(48.8)	157	(49.2)	352	(58.8)	14.22	.001	
	No	733	(47.3)	324	(51.2)	162	(50.8)	247	(41.2)			
	Spine	588	(37.9)	269	(42.5)	97	(30.4)	222	(37.1)	13.47	.001	
	No	963	(62.1)	364	(57.5)	222	(69.6)	377	(62.9)			
Disability severity^d^	Mild	1279	(82.5)	551	(87.0)	251	(78.6)	477	(79.6)	16.10	<.001	
	Severe	272	(17.5)	82	(13.0)	68	(21.4)	122	(20.4)			
ADL	Dependent	635	(40.9)	228	(36.0)	90	(28.2)	317	(52.9)	63.27	<.001	
	Independent	916	(59.1)	405	(64.0)	229	(71.8)	282	(47.1)			
IADL	Dependent	743	(47.9)	281	(44.4)	105	(32.9)	357	(59.6)	64.68	<.001	
	Independent	808	(52.1)	352	(55.6)	214	(67.1)	242	(40.4)			
Number of chronic diseases		2.39	(1.64)	2.45	(1.56)	1.83	(0.09)	2.62	(1.72)	25.99	<.001	2 < 1,3
Relationship with friends	Satisfied	1165	(75.1)	496	(78.4)	261	(81.8)	408	(68.1)	26.94	<.001	
	Dissatisfied	386	(24.9)	137	(21.6)	58	(18.2)	191	(31.9)			
Relationship with family^d^	Satisfied	1275	(82.2)	529	(83.6)	258	(80.9)	488	(81.5)	1.42	.492	
	Dissatisfied	276	(17.8)	104	(16.4)	61	(19.1)	111	(18.5)			

^a^Comparisons among the three latent classes

^b^Duncan’s test

^c^Equivalised household income

^d^Missing values included using multiple imputation

*ADL* Activities of Daily Living, *IADL* Instrumental Activities of Daily Living, *SD* Standard Deviation, *M* Mean

### Factors associated with latent class membership

Table 4 presents the results of multinomial latent class logistic regression, with the high self-management group as the reference group. Among hypertensive patients with physical disabilities, male (odds ratio [OR] 8.59, 95% confidence interval [CI] 5.48–13.45), younger (OR 0.94, CI 0.92–0.96), or currently single patients, including never married, widowed, divorced, and separated (OR 1.52, CI 1.04–2.21), were more likely to belong to the harmful habitual behaviour group. In contrast, those who were male (OR 0.46, CI 0.34–0.62) or were unemployed (OR 0.49, CI 0.36–0.65) were less likely to belong to the inactive behaviour group compared with the high self-management group. The inactive behaviour group also had severe disabilities (OR 1.78, CI 1.26–2.52), dependent status on the ADL (OR 1.48, CI 1.07–2.05), and dissatisfaction with their relationships with friends (OR 1.62, CI 1.22–2.15).

**Table 4:** Odd ratios of the association of the harmful habitual behaviour group (class 2) and the inactive behaviour group (class 3) relative to the high self-management group (class 1) based on multinomial latent class logistic regression models

Variables	Category	Harmful habitual behaviour group			Inactive behaviour group		
		OR	95% CI	*p*	OR	95% CI	*p*
Gender	Male	8.59	5.48–13.45	<.001	0.46	0.34–0.62	<.001
	(ref. Female)						
Age		0.94	0.92–0.96	<.001	1.01	0.99–1.03	.207
Education	≤ Elementary school	1.08	0.75–1.54	.684	1.09	0.82–1.45	.558
	(ref. > Elementary school)						
Current Marital status	Single	1.52	1.04–2.21	.028	0.94	0.73–1.22	.659
	(ref. Married)						
Employment status	Unemployed	0.71	0.49–1.04	.077	0.49	0.36–0.65	<.001
	(ref. Employed)						
Monthly household income	Low	0.78	0.54–1.13	.187	1.01	0.73–1.41	.916
	(ref. High)						
Disabled body part	Upper limbs	1.10	0.65–1.88	.725	0.90	0.58–1.42	.661
	(ref. No)						
	Lower limbs	1.16	0.70–1.92	.577	1.12	0.76–1.66	.558
	(ref. No)						
	Spine	1.00	0.59–1.69	.999	0.82	0.56–1.21	.315
	(ref. No)						
Disability severity^a^	Severe	1.06	0.68–1.65	.793	1.78	1.26–2.52	.001
	(ref. Mild)						
ADL	Dependent	1.29	0.82–2.01	.272	1.48	1.07–2.05	.017
	(ref. Independent)						
IADL	Dependent	1.03	0.67–1.59	.880	1.31	0.95–1.81	.103
	(ref. Independent)						
Number of chronic diseases		0.98	0.88–1.09	.705	1.00	0.93–1.08	.968
Relationship with friends	Dissatisfied	0.81	0.54–1.21	.302	1.62	1.22–2.15	.001
	(ref. Satisfied)						
Relationship with family^a^	Dissatisfied	1.13	0.72–1.77	.596	1.01	0.73–1.41	.932
	(ref. Satisfied)						

^a^Missing values included using multiple imputation

*CI* Confidence Interval, *OR* Odds Ratio, *ref* reference, *ADL* Activities of Daily Living, *IADL* Instrumental Activities of Daily Living

### Comparisons of health measures between latent class memberships

The latent classes were compared in terms of health measures, including health profile, psychological health, and patient experiences, as shown in Table 5. The mean HRQOL score in the inactive behaviour group was 0.72 ± 0.23, which was significantly lower than in the other two groups (*p* < .001). Subjective health was lowest in the inactive behaviour group (2.09 ± 0.75) and highest in the harmful habitual behaviour group (2.57 ± 0.80) (*p* < .001). There was a significant difference in self-report of depression among the groups (*p* < .006), although suicidal ideation and suicide attempts did not show any differences. The inactive behaviour group had a higher prevalence of depression (21.9%) than the other two groups. The results indicated that the harmful habitual behaviour group had the lowest score of satisfaction with healthcare services (3.54 ± 0.67; *p* < .001), while the other two groups did were comparable. Lastly, there was a significant difference in unmet medical needs among the three groups (*p* < .001). The rate of unmet medical needs was higher in the inactive behaviour group (23.7%) than in the other two groups.

**Table 5:** Differences in health profile, psychological health, and patient experience by latent classes

	Characteristics	Class^a^	M (SD) or n(%)	F or *x*^2^	*p*	Post-hoc^b^
Health profile	Health-related Quality of Life, (range − 0.171–1)	1	0.81 (1.50)	44.95	<.001	1,2 > 3
		2	0.82 (1.71)			
		3	0.72 (0.23)			
	Subjective health, (range 1–5)	1	2.40 (0.76)	46.59	<.001	2 > 1 > 3
		2	2.57 (0.80)			
		3	2.09 (0.75)			
Psychological health	Suicidal ideation	1	84 (13.3)	0.80	.671	
		2	40 (12.5)			
		3	87 (14.5)			
	Suicide attempts	1	10 (1.6)	3.40	.183	
		2	4 (1.3)			
		3	3 (0.5)			
	Depression	1	98 (15.5)	10.33	.006	
		2	49 (15.4)			
		3	131 (21.9)			
Patient experience	Satisfied with healthcare services, (range 1–5)	1	3.77 (0.68)	13.19	<.001	1,3 > 2
		2	3.54 (0.67)			
		3	3.73 (0.64)			
	Unmet medical needs	1	98 (15.5)	14.34	.001	
		2	55 (17.2)			
		3	142 (23.7)			

^a^Class 1 = high self-management group, Class 2 = harmful habitual behaviour group, Class 3 = inactive behaviour group; M mean, SD Standard Deviation

^b^Duncan's test

## Discussion

The present study has identified three mutually exclusive subgroups of hypertension self-management behaviours: a high self-management group, a harmful habitual behaviour group, and an inactive behaviour group, based on five self-management behaviours: smoking, alcohol intake, physical activity, diet, and weight control. Only 40.8% of our respondents showed adherence to the 2018 Hypertension Guidelines in their self-management of hypertension Therefore, our results extend the existing literature observations that a large proportion of patients with hypertension do not perform self-management behaviours well [7, 10, 17]. On the other hand, contrary to prior studies of people without disabilities using LCA [7, 10] a clear distinction in the self-management behaviours among groups was evident in our study. In particular, the classes exhibited stark differences with respect to physical activity (high self-management group; 100.0% vs. inactive behaviour group; 0%), although the overall probability of physical activity was not particularly different compared to previous studies [7, 10]. Since inadequate physical activity is a well-known challenge for people with physical disabilities [18], it is important to define the characteristics and health measures among the groups.

Use of tobacco and alcohol, which are strongly correlated, are known to be common health-risk behaviours that negatively impact control of high blood pressure. Within the present analysis, the harmful habitual behaviour group exhibited the lowest percentages of people avoiding smoking or excessive alcohol intake; young, single males were most likely to belong to this group. This result was in agreement with a previous study that reported frequent substance use, such as cigarettes and alcohol, is often amplified among those who are young, male, and single [19]. Young men display a greater tendency toward substance use when coping with their problems [10] and prioritise other goals before taking care of their health [20]. Conversely, female sex predicted membership in the inactive behaviour group, whose members satisfactorily complied with avoiding harmful habitual behaviours, such as tobacco and alcohol use. Such findings built on previous literature reporting that women are likely to have a lower prevalence of tobacco and alcohol use [10, 21], likely because of social and cultural factors that might discourage women from smoking and alcohol intake [19]. Accordingly, targeted policy and individual level interventions that simultaneously address tobacco use and excessive alcohol drinking could arguably be most effective when targeting addressing unhealthy behaviours in young, single males.

Regarding employment status, Rimmer [18] argued that unemployment or underemployment is related to reduced physical activity in people with disabilities. By contrast, our results indicated that being employed was associated with those in the inactive behaviour group who were physically inactive. The reasons for the inconsistency in results are uncertain, but can likely be attributed to the type of jobs that members of the disabled population tend to hold. Compared to people with other types of disabilities, those who have physical disability are more likely to have a sedentary job that requires no physical exertion. As a prior study [22] proposed that sedentary jobs can contribute to inadequate physical activity; the participants categorised in this group are highly likely to have sedentary jobs due to their physical limitations. Still, further research is warranted to explore the underlying mechanisms between health behaviours and employment among people with physical disabilities.

Dissatisfaction with friendships was another variable associated with belonging to the inactive behaviour group. Studies have provided evidence that social relationships influence health behaviours. Umberson and Karas [23] highlight that social relationships can instil a sense of responsibility and concern for others that then lead individuals to engage in positive self-care behaviours. Similarly, s*ocial isolation* is associated with less favourable lifestyle choices [24]. Notably, people who have poor functional status tend to experience a lower quality of social relationships, resulting in social isolation [25]. Coordinated programmes thereby should identify socially isolated adults with physical disabilities and mobilise local resources to offer instrumental and social support to these individuals.

Many researchers have raised concerns about the high prevalence of low-quality diet in people with disabilities [26]. This corresponds to our findings that the vast majority of the participants across the whole sample reported having an unhealthy diet, which is of high prevalence compared to the similar studies of people without disabilities [17, 27]. The low probability of achieving adequate nutrient intake was more apparent in the participants belonging to the inactive behaviour group which was associated with severe disability and ADL dependency. Among people with physical disabilities, those who are more dependent in terms of ADL may have additional functional limitations other than physical disability, such as trouble seeing or hearing, resulting in an increased risk of having a diet that may interfere with disease management protocols [28]. Thus, it is of paramount importance to identify the at-risk individuals who have poor functional abilities and urgent need for policy interventions aiming to improve diet quality among people with disabilities.

Achieving the recommended level of physical activity can be challenging for people with severe physical disabilities, particularly among those who have difficulty walking due to pain or imbalance, or those who are unable to walk due to paralysis [18]. A distinctive feature noted from the behavioural patterns in the inactive behaviour group was that none of the members engaged in regular physical activity. It can be expected the participants belonging to this group may experience substantial challenges to overcoming insufficient physical activity as they were likely to have a severe degree of physical disability [18]. Since functional level is strongly associated with physical activity, co-occurring health conditions and level of independence should be considered as important determinants of physical activity in those with physical disabilities [29]. Thus, policy and infrastructure changes to promote the inclusion of people with diverse forms and degrees of physical disabilities in physical activity initiatives are a high priority to promote physical activity and to reduce health disparity within the population with disability.

Our study further attempted to identify and differentiate previously unidentified, at-risk groups within the population of individuals with physical disabilities by comparing the health measures between the subgroups. The inactive behaviour group displayed the poorest HRQOL and subjective health and had the highest prevalence of depression and unmet medical needs. This phenomenon implies that the inactive behaviour group represents the most-at-risk population among the three groups. Accumulated evidence has demonstrated that these health measures are largely determined by various health-promoting behaviours. In particular, physical activity is emphasised as a critical behaviour that significantly impacts both immediate and long-term health [30]. Extant studies indicate that engaging in physical activity is associated with a better HRQOL and subjective health in hypertensive patients and is also effective for the treatment of depression [31–34]. Considering the fact that none of the participants in the inactive behaviour group performed physical activity, it is worth presuming that the self-management behaviour pattern in this group may have led them be at greater risk of hypertension. In other words, they exhibited a tendency to only practice certain self-management behaviours that do not require any physical capability to carry out. These findings imply that functional limitations may have acted as barriers to health-promoting activities that require physical capability, as the degree of disability and ADL dependence are closely interrelated and may have had a negative impact on their health measures.

Unmet medical needs are considered to be key determinants of health status in vulnerable populations living with disabilities and can potentially threaten the viability of community living [35]. Our results indicate that the rate of unmet medical needs in the inactive behaviour group was 23.7%, which is roughly twice as high as the 11.6% found in a previous study on Korean adults without disabilities [36]. It appears from the literature that those with the greatest need are also the least likely to have those needs met by the healthcare system [35]. Those who experience unmet medical needs are likely to practice unhealthy behaviours and experience deteriorating health conditions; this conclusion is supported by previous studies that demonstrated correlations between unmet medical needs and various proxy indicators, such as health status and death [37].

What we can conclude, as indicated in the behavioural patterns in the inactive behaviour group, is that avoiding certain harmful habitual behaviours, such as cigarette smoking and alcohol intake, alone may be insufficient to address the issues of increasing poor health risks. AlHadlq et al. [22] highlights that the cycle of good practice of self-management behaviours is interrelated and each behaviour will not work well without the other; thus, ideally these positive health behaviours should occur simultaneously. Policy makers and healthcare professionals should take into account that various components of health-promoting behaviours should be practiced in harmony. Moreover, healthcare intervention in the inactive behaviour group should be prioritised and, more importantly, it should focus on providing appropriate assistance to enhance participants’ engagement in health-promoting behaviours, especially those which require physical capability such as physical activity.

Our study has several limitations, the most severe being insufficient information to assess specific dietary patterns and obesity due to data constraints caused by using the secondary data. First, we were only able to use a single-item measure of patient-reported balanced diet adherence. Although the optimal means of assessing diet is unclear, our use of a single-item diet measure remains a limitation in that it could not capture specific dietary patterns regarding food groups and serving sizes. Future studies should use questionnaires which measure extensive diet patterns such as a food frequency questionnaire to evaluate dietary approaches to stop hypertension diet scores. Similarly, using BMI to indicate obesity was another limitation in this study. Although BMI has been used globally as a simple indicator of obesity, it is not an optimal measure of obesity in people with physical disabilities. Height and weight may not be accurate, and standard cut-offs underestimate obesity in people with physical disabilities. Thus, it is recommended that alternative anthropometric measures, such as waist circumference, which is a simple, more sensitive alternative to BMI in population with disabilities, be used instead [38]. Furthermore, due to limited information availability, we were unable to consider other variables that may be associated with hypertension management adherence such as residential area, religion, time since diagnosis of hypertension, or health literacy. The present study could not determine causal relationships due to the cross-sectional study design. Another limitation was the potential bias caused by the self-report measurement. Although the National Survey of Disabled Persons ensures the representativeness of the population with disabilities, potential threats to measurement validity cannot be adjusted. Furthermore, self-reported items such dietary assessment, are at risk of misinterpretation. Lastly, in as our analysis, the sample population had an average age of 65 or older; it remains unknown whether the same patterns occur in younger age groups.

## Conclusions

This study demonstrated the typology of hypertension self-management behavioural patterns including abstaining from tobacco use, limiting alcohol consumption, engaging in regular physical activity, eating a balanced diet, and maintaining weight control, which are essential health behaviours for the management of hypertension, as evidenced using large-scale data. The present study considers the subgrouping of hypertensive adults with physical disabilities sample into three classes: a high self-management group, a harmful habitual behaviour group, and an inactive behaviour group. Our study also identifies factors associated with hypertension self-management such as age, gender, marital status, employment, degree of physical and functional disability, and satisfaction with social relationships; it also provides important insights into how health-promotion strategies for hypertensive patients with physical disabilities might be targeted differently in each group. As such, a special consideration should be given by health professionals considering the self-management behaviours that appeared to be deficit in each group, such as avoiding habitual health-risk activities (i.e., harmful habitual behaviour group) or practicing regular physical activity (i.e., inactive behaviour group). More importantly, the at-risk population, such as the inactive behaviour group identified in our study, should be prioritised in receiving assistance with hypertension self-management behaviours that require physical capability. These observations and their implications contribute to a better understanding of this heterogeneous, underserved population and provide evidence for healthcare teams to design strategies for supporting self-management and to develop a health-promoting service model for the population with physical disabilities.

## Data Availability

The datasets generated and/or analysed in the current study are available in the repository of the Health and Welfare Data Portal, where they will be provided upon request: https://data.kihasa.re.kr/.
